# Evaluation of Gamma Radiation Properties of Four Types of Surgical Stainless Steel in the Energy Range of 17.50–25.29 keV

**DOI:** 10.3390/ma14226873

**Published:** 2021-11-15

**Authors:** Mohammad Marashdeh, Ibrahim F. Al-Hamarneh

**Affiliations:** 1 Department of Physics, College of Sciences, Imam Mohammad Ibn Saud Islamic University (IMSIU), P.O. Box 90950, Riyadh 11623, Saudi Arabia; 2 Department of Physics, Faculty of Science, Al-Balqa Applied University, Al-Salt 19117, Jordan; hamarnehibrahim@gmail.com

**Keywords:** surgical stainless steel, mass attenuation coefficient, WinXCOM, half-value layer, X-ray fluorescence

## Abstract

In this study, the gamma radiation properties of four types of surgical-grade stainless steel (304, 304L, 316 and 316L) were investigated. The effective atomic number Z${}_{eff}$, effective electron density N${}_{eff}$ and half-value layer (HVL) of four types of surgical-grade stainless steel were determined via the mass attenuation coefficient (μ/ρ). The μ/ρ coefficients were determined experimentally using an X-ray fluorescence (XRF) technique and theoretically via the WinXCOM program. The K${}_{\alpha 1}$ of XRF photons in the energy range between 17.50 and 25.29 keV was used from pure metal plates of molybdenum (Mo), palladium (Pd), silver (Ag) and tin (Sn). A comparison between the experimental and theoretical values of μ/ρ revealed that the experimental values were lower than the theoretical calculations. The relative differences between the theoretical and experimental values were found to decrease with increasing photon energy. The lowest percentage difference between the experimental and theoretical values of μ/ρ was between −6.17% and −9.76% and was obtained at a photon energy of 25.29 keV. Sample 316L showed the highest value of μ/ρ at the energies 21.20, 22.19 and 25.29 keV. In addition, the measured results of Z${}_{eff}$ and N${}_{eff}$ for all samples behaved similarly in the given energy range and were found to be in good agreement with the calculations. The equivalent atomic number (Z${}_{eff}$) of the investigated stainless-steel samples was calculated using the interpolation method to compare the samples at the same source energy. The 316L stainless steel had higher values of μ/ρ, Z${}_{eff}$ and Z${}_{eq}$ and lower values of HVL compared with the other samples. Therefore, it is concluded that the 316L sample is more effective in absorbing gamma radiation.

## 1. Introduction

Technological development in various fields has increased the quality of life of patients by improving health services. In some circumstances, medical interventions require the use of metal materials with biocompatible compositions and properties, such as stainless steel. Stainless-steel materials are used as biocompatible mineral components for rebuilding teeth, manufacturing medical devices used in surgeries and other applications [1,2]. Stainless steel is widely used in various industrial fields due to its distinctive mechanical properties and corrosion resistance. These properties are attributable to the presence of a thin surface layer rich in chromium oxyhydroxide [3]. Materials with high resistance to radiation damage, swelling and corrosion [4,5] are attractive to the nuclear industry, and stainless steel represents the best candidate with such properties [6]. Furthermore, stainless steel is used in nuclear reactors and equipment in medical and radiological research centres. Hence, investigating the radioactive behaviour and shielding properties of these materials against X-rays and gamma rays is important. Previous studies have examined the behaviour of the attenuation properties of different types of stainless materials at varying photon energies [7,8,9,10,11,12,13,14,15,16]. Low gamma-ray energies have been widely used in practical radiography and medical diagnoses, where the interaction of gamma rays depends on the photon energy and the composition of materials, that is, the atomic numbers of elements. Stainless-steel material plays a role in the manufacture of various medical tools and devices used in diagnostic and biomedical applications [17]. Commonly used stainless-steel materials in different industries are 304 [18], 304L [19], 316 [20] and 316L [21], with applications in different medical fields, such as medical and laboratory instruments and devices, especially for the manufacture of blood contact tools. Therefore, exploring the radiation properties of surgical stainless-steel materials at low photon energy is important. The ability of a material to protect against gamma radiation can be determined using several different parameters, and among the important parameters that were used in this study are the mass attenuation coefficient (μ/ρ), half-value layer (HVL), effective atomic number (Z${}_{eff}$), effective electron density (N${}_{eff}$) and equivalent atomic number (Z${}_{eq}$) in the energy range of 17.50–25.29 keV.

## 2. Materials and Methods

### 2.1. Theory

Four different samples of stainless steel were studied in this work. The chemical compositions and densities of the stainless steel used in this study are shown in Table 1.

The main parameter of photon penetration in samples is the mass attenuation coefficient $\mu_{m}$, which is calculated using Equation (1) [23]:

(1) $$\begin{array}{c} I=I_{o}e^{-\mu_{m}x} \end{array}$$

where $I_{0}$ and I are the incident and transmitted gamma rays, respectively, and x is the mass thickness of a stainless-steel material.

Equation (2) shows the maximum total standard error in mass attenuation coefficients $\Delta (\mu_{m})$.

(2) $$\begin{array}{c} \Delta \left(\mu_{m}\right)=\frac{1}{x}\sqrt{{\left(\frac{\Delta I_{o}}{I_{o}}\right)}^{2}+{\left(\frac{\Delta I}{I}\right)}^{2}+{\left(\frac{\Delta I_{o}}{I_{o}}\right)}^{2}{\left(\frac{\Delta x}{x}\right)}^{2}} \end{array}$$

where $\Delta I_{o}$, ΔI and Δx are the standard errors in the intensities of $I_{o}$, I and the mass thickness x, respectively. The theoretical mass attenuation coefficient was calculated using WinXCOM, a database that contains photon cross-section data for components of materials. The values of mass attenuation coefficients can lead to the determination of the total atomic cross-section, which can be determined using Equation (3) [23]:

(3) $$\begin{array}{c} \sigma_{a}=\frac{{\left(\mu_{m}\right)}_{sample}}{N_{A}\sum_{i}^{n}\left(W_{i}/A_{i}\right)} \end{array}$$

where $N_{A}$ is Avogadro’s number, and $A_{i}$ is the atomic weight of a constituent element of the sample. The total electronic cross-section for the element is given by Equation (4) [23]:

(4) $$\begin{array}{c} \sigma_{el}=\frac{1}{N_{A}}\sum_{i}^{n}\frac{f_{i}A_{i}}{Z_{i}}{\left(\mu_{m}\right)}_{i} \end{array}$$

where $f_{i}$ is the number of atoms of element *i* relative to the total number of atoms of all elements in the alloy, and $Z_{i}$ is the atomic number of the *i*th element in the alloy.

The effective atomic number ($Z_{eff}$) and effective electron number ($N_{eff}$) are very important parameters when it comes to choosing a suitable material for radiation dosimetry and detection. The effective atomic number ($Z_{eff}$) of the compound can be found from the ratio between the total atomic cross-section and the total electronic cross-section through Equation (5) [23], and the effective electron number ($N_{eff}$) is evaluated as shown in Equation (6).

(5) $$\begin{array}{c} Z_{eff}=\frac{\sigma_{a}}{\sigma_{al}} \end{array}$$

(6) $$\begin{array}{c} N_{eff}=\frac{\mu_{m}}{\sigma_{al}} \end{array}$$

The half-value layer (HVL) is the thickness of a material that reduces the radiation level by a factor of 2, which can be described by Equation (7). HVL is very important in radiation-related investigations because it predicts the thickness required to achieve any radiation shielding.

(7) $$\begin{array}{c} HVL=\frac{ln2}{\mu } \end{array}$$

Moreover, the equivalent atomic number ($Z_{eff}$), which provides a description of the properties of the investigated material in terms of its equivalent elements, can be calculated by applying the logarithmic interpolation method. In this method, the Compton partial mass attenuation coefficient, ($\mu_{m}$)${}_{comp}$, and the total mass attenuation coefficient, ($\mu_{m}$)${}_{Total}$, for the studied material and two other adjacent elements having known atomic numbers $Z_{1}$ and $Z_{2}$ must first be determined at a certain energy. Then, $Z_{eq}$ of the material can be calculated by means of the following formula (Equation (8)) [24,25]:

(8) $$\begin{array}{c} Z_{eff}=\frac{Z_{1}\left(logR_{2}-logR\right)-Z_{2}\left(logR-logR_{1}\right)}{logR_{2}-logR_{1}} \end{array}$$

where $R_{1}$ and $R_{2}$ are the ${\left(\mu_{m}\right)}_{comp}/{\left(\mu_{m}\right)}_{Total}$ ratios corresponding to elements with atomic numbers $Z_{1}$ and $Z_{2}$, respectively, and R is the ${\left(\mu_{m}\right)}_{comp}/{\left(\mu_{m}\right)}_{Total}$ ratio for the investigated stainless-steel material, which lies between the ratios $R_{1}$ and $R_{2}$.

### 2.2. Experimental Setup

**Figure 1:**
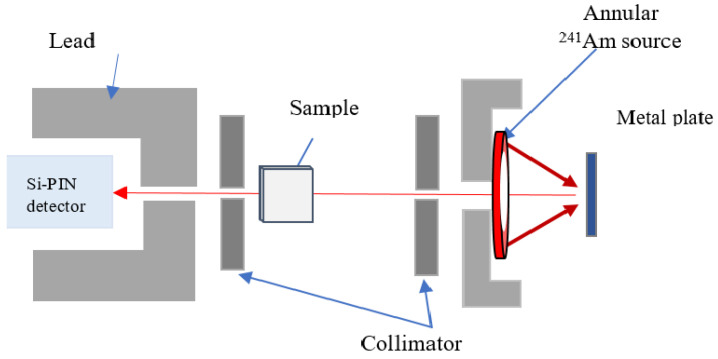
Experimental XRF setup.

The linear and mass attenuation coefficients were evaluated by using an X-ray fluorescent (XRF) system. The XRF energies produced from pure metal plates were irradiated to the annular source of radioactive ${}^{241}Am$ with 3.7 GBq activity. The pure metal plates used in this experiment were molybdenum (Mo), palladium (Pd), silver (Ag) and tin (Sn) with photon energies of 17.5, 21.2, 22.19 and 25.29 keV, respectively. The details of the experimental setup are shown in Figure 1. The same principle of the XRF system was used in previous work [26,27]. A Si-PIN photodiode XR-100 CR detector with an active area of 7 mm${}^{2}$ and thickness of 300 µm was used to detect the intensities of X-ray fluorescence (XRF) energies. Samples were irradiated for 7200 s to ensure the reliability of the results. A collimator with a diameter of 0.5 cm was used in the Si-PIN detector to avoid the detection of any scattering and background radiation. The distance between the pure metal plate and the sample was 16.2 cm, and that between the sample and the detector was 13.1 cm. These distances were chosen based on the final adjustment procedure using a gamma-ray source and monitoring the beam at the detector collimator. This procedure was performed several times using different distances between the metal plate and sample and between the sample and detector to ensure that the beam was aligned with all parameters of the XRF system. Figure 1 shows the experimental setup used in this work. Samples 304 and 304L with thicknesses of 0.4, 0.6 and 1.0 mm and samples 316 and 316L with thicknesses of 0.5, 0.8 and 1.5 mm were utilised in this study. The region of interest for K${}_{\alpha 1}$ peak energies was evaluated from the results of the spectrum of pure metal plates. Then, the net area value was recorded as *I* and $I_{o}$ for the attenuated and unattenuated XRF spectra of the selected metal plates, respectively. The linear attenuation coefficient was obtained by the slope of the resulting line by plotting $ln(I_{o}/I)$ against the thickness of the stainless-steel samples.

## 3. Results

### 3.1. Mass Attenuation Coefficient

The mass attenuation coefficients of surgical stainless-steel samples obtained in the photon energy range of 17.50–25.29 keV using XRF beams from Mo, Pd, Ag and Sn are listed in Table 2. The measurement error of $\mu_{m}$ using XRF energies was between 0.95% and 2.30%. The error rate was slightly higher at a photon energy of 17.50 keV than that at other photon energies. Intensities of incident and transmitted XRF beams were determined using the net count of the $k_{\alpha 1}$ peak. The results show that $\mu_{m}$ decreased rapidly with the increase in photon energy. The values of $\mu_{m}$ ranged from 17.5 to 25.29 keV. At these energies, the dominant interaction between photon beams and stainless-steel samples, which depends largely on the photon energy and the elemental composition of samples, is photoelectric absorption.

Table 3 shows that the experimentally calculated attenuation coefficient values were lower than the theoretical results obtained using WinXCOM; the percentage difference between experimental and theoretical $\mu_{m}$ for all samples was between 6.17% and 15.61%. This difference was particularly evident at certain photon energies, such as 17.5 keV, at which the percentage difference between experimental and theoretical values was −12.72–15.61%, which was larger than that at other energies. The percentage difference decreased with the increase in photon energy. All samples obtained a percentage difference between −6.17% and −9.76% at an energy of 25.29 kV, likely due to the presence of some impurities in surgical stainless-steel samples, in addition to some errors from the experimental measurement process through the XRF system.

**Figure 2:**
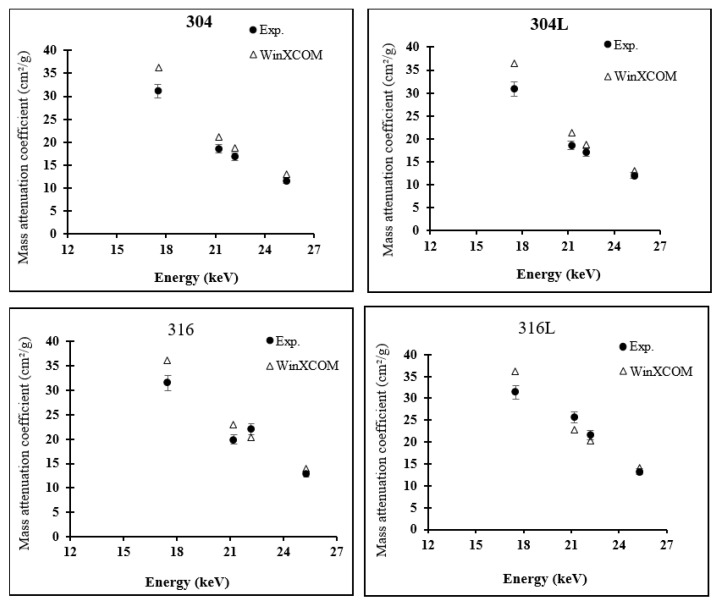
Comparison of mass attenuation coefficients for all four stainless-steel samples evaluated experimentally and calculated theoretically using WinXCOM.

Figure 2 shows the behaviour of $\mu_{m}$ of samples at an energy range of 17.50–25.29 keV. For all samples, experimental $\mu_{m}$ was consistent with theoretical values at energies of 21.20, 22.19 and 25.29 keV. Sample 316L obtained the maximum $\mu_{m}$ value at these energies. Hence, sample 316L is recommended for use in diagnostic radiology equipment because it meets the photon energy range requirement. The experimental $\mu_{m}$ values for stainless-steel samples were close to one another at an energy of 17.50 keV. However, theoretical and experimental values were inconsistent at 17.50 keV; the experimental results showed that material 316 had the largest $\mu_{m}$, whereas the theoretical results showed that material 304L had the highest $\mu_{m}$ value of 36.58 cm²/g. This finding suggests that samples with energies less than 17.50 keV require further investigation.

### 3.2. Effective Atomic Number ($Z_{eff}$) and Electron Density ($N_{eff}$)

$Z_{eff}$ and Neff values for the surgical stainless-steel samples in this work were evaluated using the mass attenuation coefficient, which was calculated from the experimental and theoretical (via WinXCOM) results. The values of $Z_{eff}$ and $N_{eff}$ for different samples of stainless steel are listed in Table 4. The theoretical $Z_{eff}$ and $N_{eff}$ values (WinXCOM) of samples slightly increased with the increase in photon energy. The experimental $Z_{eff}$ and $N_{eff}$ values of the samples appeared to have more variation among energies compared to theoretical values, but the theoretical and experimental results were consistent in terms of materials 316 and 316L obtaining the maximum value of $Z_{eff}$ at different energies compared to the other materials. This finding shows that materials 316 and 316L are slightly better at shielding compared with materials 304 and 304L and can be used in shielding devices in medical and radiological applications that use energies that range between 17.50 and 25.29 keV. The $N_{eff}$ results for selected stainless-steel samples, which presented the same behaviour as $Z_{eff}$ values, are also listed in Table 4.

### 3.3. Half-Value Layer (HVL)

**Figure 3:**
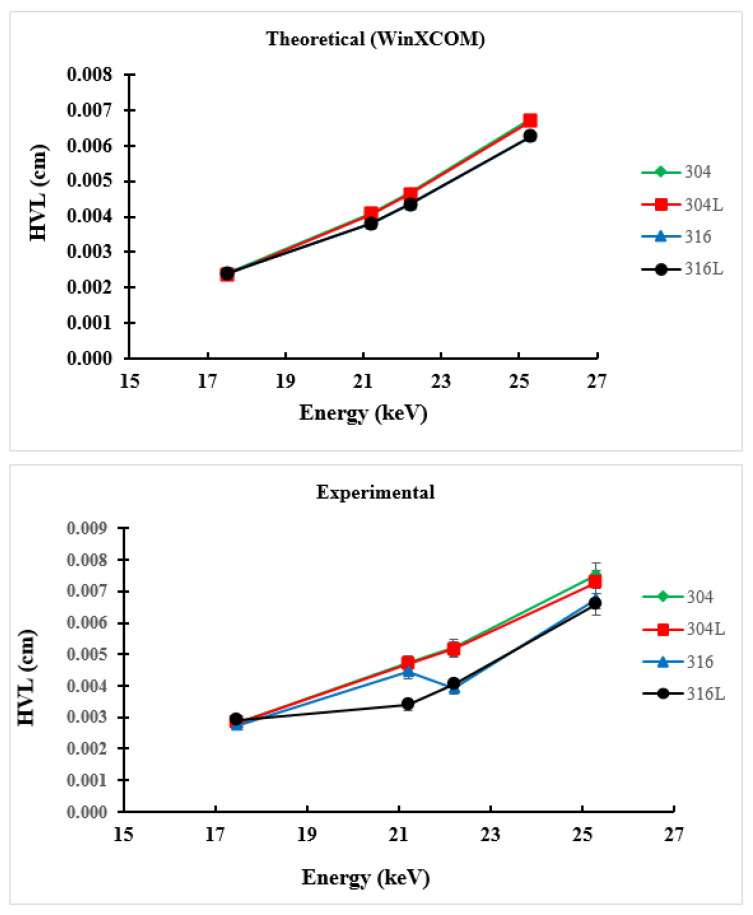
Variation in theoretical and experimental HVL for 304, 304L, 316 and 316L stainless-steel materials with photon energy in the range 17.5–25.29 keV.

HVL is an important parameter in the design of radiation protection and refers to the thickness of the sample required to absorb half the value of the radiation falling on it. The results of theoretically and experimentally calculated HVL values are presented in Figure 3. The value of HVL increased with the increase in photon energy. In addition, the HVL values of surgical stainless-steel samples determined experimentally and calculated using WinXCOM were close to one another, with slight differences. Figure 3 clearly shows minimal differences between experimental and theoretical HVL values among samples, among which sample 316L obtained the lowest value. This is due to some differences in the proportions of elemental components in the steel materials. A low HVL value indicates a high ability to absorb radiation [28]; hence, the material can be used as a shield for gamma-ray radiation.

### 3.4. Equivalent Atomic Number ($Z_{eq}$) for the Investigated Surgical Stainless Steel

**Figure 4:**
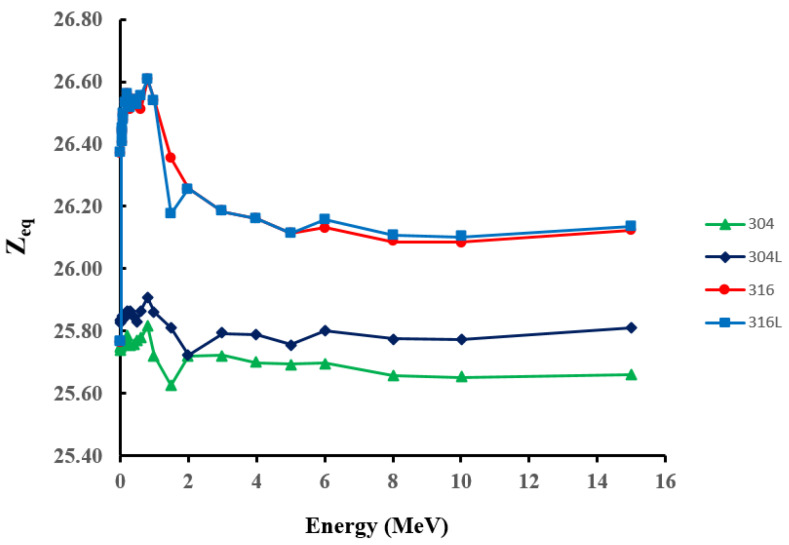
Equivalent atomic number ($Z_{eq}$) results for the studied stainless-steel samples in the energy range 0.015–15 MeV.

$Z_{eq}$ was evaluated based on the calculated $\mu_{m}$ values described above for the studied surgical stainless-steel materials in the energy range 0.015–15 MeV. The results are shown in Figure 4. The results show that $Z_{eq}$ demonstrated the same energy behaviour for all samples. For all samples, $Z_{eq}$ tended to reach its maximum value at intermediate energies (0.6–1 MeV) and then decrease to slightly lower values as energy increased to the pair-production dominance regime. Moreover, the values of surgical stainless-steel types 316 and 316L were almost the same and slightly higher than those of surgical-stainless steel types 304 and 304L for the whole energy range. These variations in $Z_{eq}$ values may be attributed to variations in the weight fraction of the constituent elements in steel materials. In particular, elements with high z numbers, Ni and Mo, are only present in the 316 and 316L steel materials, as shown in Table 1. The best shielding properties are characterised by higher $Z_{eq}$ values. Therefore, it can be concluded that 316 and 316L samples can be considered to be more effective in shielding against radiation than 304 and 304L samples.

## 4. Conclusions

An experimental XRF system and the WinXCOM program were used to evaluate the mass attenuation coefficient ($\mu_{m}$), effective atomic number ($Z_{eff}$), electron density ($N_{eff}$) and HVL of several surgical stainless-steel materials in the photon energy range of 17.50–25.29 keV. The behaviour of $\mu_{m}$ was generally consistent between experimental and theoretical (via WinXCOM) results, with materials 316 and 316L presenting the highest value of $\mu_{m}$ in the energy range of 17.50–25.29 keV compared with materials 304 and 304L. In addition, a comparison of experimental and theoretical $\mu_{m}$ values showed a percentage difference between −12.72% and 15.61% at a photon energy of 17.50 keV, while this difference decreased as energy increased to 25.29 keV. This finding indicates that the composition of the stainless-steel material plays an important role in calculating the attenuation coefficient in a photon energy range between 17.50 and 25.29 keV. The $Z_{eff}$ and $N_{eff}$ results for stainless-steel samples behaved similarly in the studied photon energy range. Moreover, HVL values for stainless-steel samples increased as the photon energy increased. The HVL and $Z_{eq}$ results for stainless-steel samples provide more evidence that samples 316 and 316L have better shielding properties than the other stainless-steel samples considered in this study. It is expected that the presented information on surgical stainless-steel samples (304, 304L, 316 and 316L) in this study will be useful for other shielding, radiological and medical physics studies, as these materials have not been previously studied in the reported range of photon energies.

## Figures and Tables

**Table 1:** Elemental composition by relative weight and relative density of the stainless-steel materials used in this work.

Element	Material			
	304 [22]	304L [22]	316 [22]	316L [22]
C	0.07	0.03	0.08	0.03
Si	0.75	0.75	0.75	0.75
Mn	2.0	2.0	2.0	2.0
Cr	18.5	18.5	17	17
Ni	9.25	10	12	12
Mo	-	-	2.5	2.5
P	0.045	0.045	0.045	0.045
S	0.03	0.03	0.03	0.03
N	0.10	0.10	0.10	0.10
Fe	Balance	Balance	Balance	Balance
Relative density (g/cm${}^{3}$)	7.93	7.93	7.98	7.98

**Table 2:** Measured linear and mass attenuation coefficients of surgical stainless-steel samples.

Samples	Mo (17.50 keV)			Pd (21.20 keV)			Ag (22.19 keV)			Sn (25.29 keV)		
	μ	$\mu_{m}$	**Error (±%)**	μ	$\mu_{m}$	**Error (±%)**	μ	$\mu_{m}$	**Error (±%)**	μ	$\mu_{m}$	**Error (±%)**
304	246.78	31.12	1.74	146.70	18.50	1.53	133.06	16.78	1.27	92.24	11.63	1.07
304L	244.80	30.87	1.85	147.34	18.58	1.49	134.48	16.96	1.00	95.24	12.01	1.07
316	251.57	31.53	2.06	155.55	19.95	1.28	176.22	22.08	1.36	102.45	12.84	1.58
316L	239.05	31.40	2.30	204.18	25.59	1.92	172.15	21.57	1.55	105.20	13.18	0.95

**Table 3:** Mass attenuation coefficients (cm${}^{2}$/g) of 304, 304L, 316 and 316L stainless-steel samples measured experimentally and compared with theoretical values using WinXCOM.

Material	Energy (keV)	$\mu_{m}$ (cm${}^{2}$/g)		
		**Experimental**	**Theoretical (WinXCOM)**	**Percentage Deviation %**
304	17.50	31.12	36.24	−14.13
	21.20	18.50	21.2	−12.74
	22.19	16.78	18.65	−10.03
	25.29	11.63	12.89	−9.76
304L	17.50	30.87	36.58	−15.61
	21.20	18.58	21.41	−13.22
	22.19	16.96	18.83	−9.94
	25.29	12.01	13.02	−7.76
316	17.50	31.53	36.12	−12.72
	21.20	19.95	22.94	−13.03
	22.19	22.08	20.28	8.89
	25.29	12.84	14.04	−8.56
316L	17.50	31.40	36.14	−13.11
	21.20	25.59	22.95	11.49
	22.19	21.57	20.29	6.32
	25.29	13.18	14.05	−6.17

**Table 4:** The effective atomic number and effective electron density of the stainless-steel samples.

Material	Energy (keV)	$Z_{\mathrm{eff}}$		$N_{\mathrm{eff}}$ (×10${}^{23}$ Electrons g${}^{-1}$)	
		**Experimental**	**Theoretical (WinXCOM)**	**Experimental**	**Theoretical (WinXCOM)**
304	17.50	21.95	25.56	2.40	2.79
	21.20	22.30	25.55	2.44	2.79
	22.19	23.03	25.57	2.52	2.80
	25.29	23.06	25.55	2.52	2.79
304L	17.50	21.69	25.69	2.36	2.80
	21.20	22.30	25.70	2.43	2.80
	22.19	23.16	25.71	2.52	2.80
	25.29	23.71	25.71	2.58	2.80
316	17.50	22.77	26.07	2.44	2.79
	21.20	22.92	26.36	2.46	2.82
	22.19	28.82	26.47	3.09	2.84
	25.29	24.17	26.43	2.59	2.83
316L	17.50	22.67	26.09	2.43	2.80
	21.20	29.40	26.37	3.15	2.83
	22.19	28.16	26.49	3.02	2.84
	25.29	24.81	26.45	2.66	2.83

## Data Availability

Not applicable.

## References

[1] Helsen J.A., Jürgen Breme H. (1998). Metals as Biomaterials.

[2] Tandon R. (1999). Net-shaping of Co-Cr-Mo (F-75) via metal injection molding. Cobalt-Base Alloys for Biomedical Applications.

[3] Loto R.T. (2017). Study of the corrosion behaviour of S32101 duplex and 410 martensitic stainless steel for application in oil refinery distillation systems. J. Mater. Res. Technol..

[4] Daghbouj N., Li B., Callisti M., Sen H., Karlik M., Polcar T. (2019). Microstructural evolution of helium-irradiated 6H–SiC subjected to different irradiation conditions and annealing temperatures: A multiple characterization study. Acta Mater..

[5] Daghbouj N., Li B., Callisti M., Sen H., Lin J., Ou X., Karlik M., Polcar T. (2020). The structural evolution of light-ion implanted 6H-SiC single crystal: Comparison of the effect of helium and hydrogen. Acta Mater..

[6] Li B., Liao Q., Zhang H., Shen T., Ge F., Daghbouj N. (2021). The effects of stress on corrosion behavior of SIMP martensitic steel in static liquid lead-bismuth eutectic. Corros. Sci..

[7] Singh R., Singh S., Singh G., Thind K.S. (2017). Gamma radiation shielding properties of steel and iron slags. New J. Glass Ceram..

[8] Çalık A., Akbunar Ş., Uçar N., Yılmaz N., Karakaş M.S., Akkurt İ. (2014). A comparison of radiation shielding of stainless steel with different magnetic properties. Nucl. Technol. Radiat. Prot..

[9] Buyuk B. (2015). Gamma attenuation behavior of some stainless and boron steels. Acta Phys. Pol. A.

[10] Singh V.P., Medhat M., Shirmardi S. (2015). Comparative studies on shielding properties of some steel alloys using Geant4, MCNP, WinXCOM and experimental results. Radiat. Phys. Chem..

[11] Aygün B., Şakar E., Korkut T., Sayyed M., Karabulut A., Zaid M. (2019). Fabrication of Ni, Cr, W reinforced new high alloyed stainless steels for radiation shielding applications. Results Phys..

[12] Manjunatha H., Seenappa L. (2019). Gamma and X-ray Shielding Properties of Various Types of Steels. J. Nucl. Eng. Radiat. Sci..

[13] Akkurt I., Akyıldırım H., Calik A., Aytar O., Uçar N. (2011). Gamma ray attenuation coefficient of microalloyed stainless steel. Arab. J. Sci. Eng..

[14] Eissa M., Salama E., El-Kameesy S., Tageldin A. (2018). Gamma Rays Attenuation Properties of Tungsten Stainless Steel Alloys. Arab J. Nucl. Sci. Appl. (Online).

[15] Aygün B. (2020). High alloyed new stainless steel shielding material for gamma and fast neutron radiation. Nucl. Eng. Technol..

[16] Singh V., Badiger N. (2013). Study of mass attenuation coefficients, effective atomic numbers and electron densities of carbon steel and stainless steels. Radioprotection.

[17] Dehghan-Manshadi A., Yu P., Dargusch M., StJohn D., Qian M. (2020). Metal injection moulding of surgical tools, biomaterials and medical devices: A review. Powder Technol..

[18] Khalili E., Sarafbidabad M. (2017). Combination of laser patterning and nano PTFE sputtering for the creation a super-hydrophobic surface on 304 stainless steel in medical applications. Surf. Interfaces.

[19] Bait L., Azzouz L., Madaoui N., Saoula N. (2017). Influence of substrate bias voltage on the properties of TiO2 deposited by radio-frequency magnetron sputtering on 304L for biomaterials applications. Appl. Surf. Sci..

[20] Haidopoulos M., Turgeon S., Sarra-Bournet C., Laroche G., Mantovani D. (2006). Development of an optimized electrochemical process for subsequent coating of 316 stainless steel for stent applications. J. Mater. Sci. Mater. Med..

[21] Bandar A.M., Vo P., Mongrain R., Irissou E., Yue S. (2014). Effect of heat treatment on the microstructure and mechanical properties of stainless steel 316L coatings produced by cold spray for biomedical applications. J. Therm. Spray Technol..

[22] Steels A. (2013). Stainless Steel Grade Datasheets.

[23] Hubbell J.H. (1982). Photon mass attenuation and energy-absorption coefficients. Int. J. Appl. Radiat. Isot..

[24] Harima Y. (1983). An approximation of gamma-ray buildup factors by modified geometrical progression. Nucl. Sci. Eng..

[25] Elsafi M., Alrashedi M., Sayyed M., Al-Hamarneh I.F., El-Nahal M., El-Khatib M., Khandaker M.U., Osman H., Askary A.E. (2021). The Potentials of Egyptian and Indian Granites for Protection of Ionizing Radiation. Materials.

[26] Marashdeh M., Bauk S., Tajuddin A., Hashim R. (2012). Measurement of mass attenuation coefficients of Rhizophora spp. binderless particleboards in the 16.59–25.26 keV photon energy range and their density profile using x-ray computed tomography. Appl. Radiat. Isot..

[27] Akhdar H., Marashdeh M., AlAqeel M. (2021). Investigation of gamma radiation shielding properties of polyethylene glycol in the energy range from 8.67 to 23.19 keV. Nucl. Eng. Technol..

[28] Lakshminarayana G., Kumar A., Dong M., Sayyed M., Long N.V., Mahdi M. (2018). Exploration of gamma radiation shielding features for titanate bismuth borotellurite glasses using relevant software program and Monte Carlo simulation code. J. Non-Cryst. Solids.

